# Effect of Gastrointestinal Helminths and Haematological Parameters on Flea Abundance in Small Mammals in Tanzania

**DOI:** 10.1155/japr/3143283

**Published:** 2026-11-01

**Authors:** Adrian E. Materu, Eliakunda M. Mafie, Jahashi S. Nzalawahe, Ladslaus L. Mnyone

**Affiliations:** ^1^ Department of Microbiology Parasitology and Biotechnology College of Veterinary Medicine and Biomedical Sciences Chuo Kikuu Sokoine University of Agriculture Morogoro Tanzania suanet.ac.tz; ^2^ Africa Centre of Excellence for Innovative Rodent Pest Management and Biosensor Technology Development Institute of Pest Management Chuo Kikuu Sokoine University of Agriculture Morogoro Tanzania suanet.ac.tz; ^3^ Division of Science Technology and Innovation Ministry of Education Science and Technology Dodoma Tanzania moe.gov.sa

**Keywords:** fleas, gastrointestinal helminths, haemoglobin, packed cell volume, small mammals, Tanzania

## Abstract

Fleas are important vectors of numerous pathogens, including bacteria, viruses, helminths and protozoa, and their abundance on small mammals may be influenced by various biotic and abiotic factors. This study investigated the associations between gastrointestinal helminth infections, selected haematological parameters and flea abundance in small mammals from three selected districts of Tanzania. Two wards and two villages within each district were purposively selected for sampling in Mbulu, Lushoto and Morogoro District Council. Small mammals were captured from households, fallow lands, crop fields, plantation forests and bush habitats and identified to species or genus level using morphological keys. Fleas, blood samples and gastrointestinal tracts were collected to determine flea infestation, gastrointestinal helminth infections, packed cell volume (PCV) and haemoglobin concentration (Hb). Associations between gastrointestinal helminths, haematological parameters and flea abundance were assessed using generalised linear mixed models. A total of 302 rodents and shrews were captured, with *Mastomys natalensis* (54.4%, *n* = 163), *Rattus rattus* (13.6%, *n* = 41) and *Crocidura* spp. (10.3%, *n* = 31) being the most abundant hosts. Overall gastrointestinal helminth prevalence was 71.2% (95% CI: 65.7–76.2), with *Trichuris* spp. (33.3%, *n* = 100), *Hymenolepis diminuta* (29.1%, *n* = 88), *Angiostrongylus* spp. (19.9%, *n* = 60) and *Hymenolepis nana* (15.2%, *n* = 46) being the most prevalent helminths. A total of 271 fleas were collected, predominantly *Ctenophthalmus* spp. (31.0%, *n* = 84), *Pulex irritans* (30.3%, *n* = 82), *Dinopsyllus lypusus* (28.8%, *n* = 78) and *Nosopsyllus incisus* (4.1%, *n* = 11). Overall gastrointestinal helminth prevalence was significantly associated with total flea abundance (*p* < 0.05), whereas PCV and Hb showed no significant associations (*p* > 0.05). These findings suggest that coinfections involving gastrointestinal helminths and fleas are common in small mammals and that helminth infections may influence flea abundance. Understanding these host–parasite interactions may improve our understanding of pathogen transmission dynamics and inform surveillance strategies for zoonotic diseases in Tanzania.

## 1. Introduction

Small mammals are among the most abundant and diverse mammalian groups and serve as important reservoirs of pathogens, endoparasites and ectoparasites [1]. Rodents constitute the majority of small mammal species globally. In Tanzania, they account for more than 28% of all mammalian species and are widely distributed across the country [2, 3]. Their high diversity and abundance are attributed to ecological adaptability, broad dietary preferences, short life cycles and high reproductive rates [4–6]. Shrews represent another important group of insectivorous small mammals, with over 40% of global species occurring in Africa. Importantly, both rodents and shrews act as reservoirs of diverse zoonotic pathogens and play a key role in the transmission of viruses, bacteria, fungi and rickettsiae [7–9]. Transmission of these pathogens is often mediated by ectoparasites, many of which are blood‐feeding and capable of infecting both animals and humans [7].

Fleas are among the most important rodent‐associated ectoparasites and are capable of transmitting a wide range of pathogens, including bacteria, viruses, rickettsiae, protozoa and helminths [1, 10]. Flea abundance on small mammals is influenced by both biotic and abiotic factors [1, 11, 12], including environmental conditions such as temperature and humidity, as well as host‐related traits such as immunity, sex and age [11]. In addition, interactions among parasite species may influence infection dynamics, a phenomenon referred to as polyparasitism [13]. The presence of one ectoparasite taxon may facilitate infestation by others, and helminth infections may similarly influence ectoparasite burdens through host‐mediated effects.

Haematological parameters have also been reported to vary in response to parasitic infections and infestations [14, 15]. Fleas are obligate haematophagous ectoparasites that depend on host blood for survival and reproduction [16]. Consequently, host blood quality, particularly its nutritional status, may influence ectoparasite abundance and fitness. Packed cell volume (PCV) and haemoglobin concentration (Hb) are commonly used indicators of host blood quality [15]. Although some vectors exhibit preferences for hosts with particular haematological profiles, substantial variation exists within and between hosts [13, 15]. Variations in haematological traits have been shown to affect blood‐feeding success in vectors such as mosquitoes, potentially influencing pathogen transmission and ectoparasite distribution patterns [14, 15].

In Tanzania, studies examining host–parasite interactions, particularly the co‐occurrence of ectoparasites and endoparasites in small mammals, remain limited and geographically fragmented. Most available studies have focused on a few regions and predominantly on gastrointestinal helminths of *Mastomys natalensis*, with limited attention to other small mammal species such as *Gerbilliscus vicinus* [12, 17, 18]. To address this gap, the present study investigated the relationship between gastrointestinal helminth infections, haematological parameters and flea abundance in small mammals from selected plague‐endemic and nonendemic areas of Tanzania. The study provides insights into the relationships between gastrointestinal helminth infections, flea abundance and selected haematological parameters in small mammals in selected areas of Tanzania.

## 2. Materials and Methods

### 2.1. Study Area

This study was conducted in three districts namely Lushoto in Tanga Region, Mbulu in Manyara Region and Morogoro Rural in Morogoro Region, from March to July 2022. Lushoto and Mbulu districts are endemic for plague, whereas Morogoro Rural district is nonendemic. Lushoto district is located between 4°32 ^′^ S and 38°37 ^′^ E and lies mostly within the Western Usambara Mountains, which forms a part of the Eastern Arc Mountains. Lushoto is one of the major plague endemic foci and it experienced outbreaks from 1980 to 2003 [19]. Mbulu district is located between 03° 51 ^′^ S and 35°32 ^′^ E and is another main plague‐active foci in Tanzania. It experienced the last plague outbreak in 2010, after about 32 years since last outbreak [20]. Morogoro Rural district is situated between 06° 54 ^′^ S and 37°54 ^′^ E, and no plague outbreak has ever been recorded in the area. In the above regions, the wet season occurs from November to May, followed by the dry season from June to October. The sampling period coincided with the wet season in both regions.

Two wards and two villages in each were purposively selected and sampled per district. Study villages in endemic districts were selected based on previous history of plague outbreaks and likelihood of high rodent population whereas study villages in nonendemic district were selected based on convenience and likelihood of high rodent population. In Lushoto, the study villages were Mavumo and Gologolo at Shume ward, as well as Madala and Manolo at Manolo ward. In Mbulu, the study villages were Haysali and Nahasey at Nahasey ward as well as Hayaseng and Arri at Yaeda Ampa ward. In Morogoro Rural, the study villages were namely Kisaki Kituoni and Igomelo, Kisaki ward as well as Bonye and Mbwade, Bwakila Chini ward (Figure 1).

**Figure 1:**
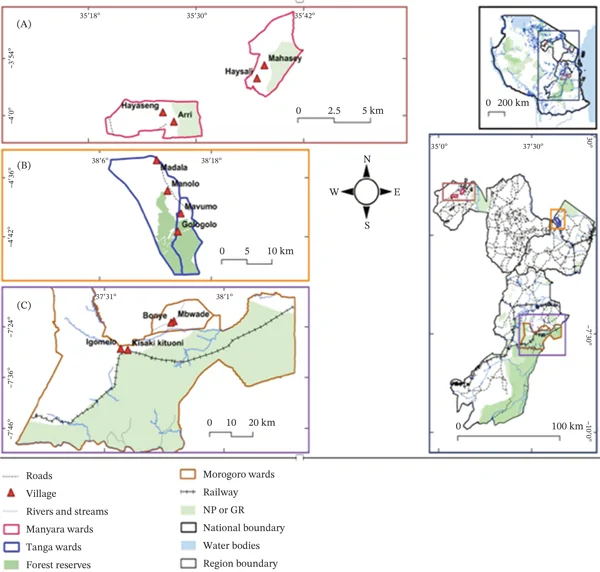
The map of (A) Mbulu district, (B) Lushoto district and (C) Morogoro Rural district showing wards and villages in which the study was conducted.

### 2.2. Study Design, Trapping and Identification of Small Mammals

Small mammals were collected using Sherman live traps baited with mixture of peanut butter and maize flour/roasted sardines. A total of 60 Sherman live traps were placed on the desired outdoor habitat type for overnight, whereas only 3 traps were deployed per household. The trapping was done for three consecutive days in each village, making it 6 days of sampling per ward. The identification of rodents and shrews as well as collection of their associated ectoparasites was done in the field. Prior to identification and collection of ectoparasites, rodents and shrews were anesthetized, one by one, inside a jar with cotton wool soaked in 98% diethyl ether [21, 22]. The rodents and shrews were identified using morphological keys [23, 24] and with assistance from experienced experts at the Institute of Pest management, Sokoine University of Agriculture‐Tanzania. Weight, sex, head body length, tail length, ear length and hindleg foot length were also measured for aiding small mammal identification.

### 2.3. Sample Collection, Processing and Identification of Fleas and Gastrointestinal Helminths

To collect fleas and other ectoparasites, anaesthetized individual rodent/shrew was combed using a fine shoe‐brush whilst inside the plastic basin. After collection, the fleas, ticks and mites were stored in vials with 70% ethanol. Fleas were processed using slightly modified procedures described by Kilonzo [25] and identified to genus or species using morphological keys described by Kilonzo [25], Segerman [26] and Harimalala et al. [27].

Rodents were dissected, gastrointestinal tracts extracted aseptically, placed on 70% ethanol within well labelled falcon tubes and transported for further laboratory analysis. The preserved gastrointestinal tracts were withdrawn individually from the falcon tubes, washed thoroughly with normal saline in petri dish and dissected longitudinally to inspect the presence of any adult, or part of adult helminths. The walls of intestines were gently scrapped bluntly to ensure all the parasites have been harvested. The adult helminths were placed in 70% ethanol, the isolated helminth were cleared with lactophenol, mounted using Canada balsam, and subsequently identified according per morphological keys by Yamaguti [28] and Soulsby [29]. The mixture of gastrointestinal contents and normal saline (0.9% NaCl) was also examined under stereo‐ and compound‐microscope to identify helminth eggs and or larvae [29, 30].

### 2.4. Assessment of Haematological Parameters

Blood was drawn from captured rodents and shrews through heart puncture into vacutainers containing sodium ethylene diamine tetraacetate (EDTA). PCV and Hb were measured from a 10‐*μ*L sample aliquoted into a standard disposable microcuvette that was placed on and measured in automatic haematology analyser (Compteur analyseur d′hematologie MS4‐s).

### 2.5. Data Analysis

The raw data were cleaned and double entered on Microsoft Excel 2007. Descriptive data were summarised in tables and graphs. A total of 302 rodents were examined for flea infestation and gastrointestinal helminths. Of these, 247 rodents from which sufficient blood samples were obtained were included in the assessment of PCV and Hb levels. All data were tested for normality and none of the data were normally distributed (Shapiro–Wilk test, *p* < 0.05). The PCV and Hb values were tested for variation within host species by nonparametric test, Kruskal–Wallis test. The prevalence of gastrointestinal helminths was calculated per the procedure described in Bush et al. [31]. A small mammal was considered infected when at least one helminth stage (egg, larva or adult) was detected in the gastrointestinal tract. Prevalence was calculated as the number of infected rodents divided by the total number of rodents examined, expressed as a percentage. The prevalence of gastrointestinal helminths was compared across host species, sex, habitat of collection and locality by chi‐square test. Generalised linear mixed model (GLMM) was used to determine association between helminths prevalence, PCV and Hb to the dependent variable, flea mean abundance. Total flea abundance and individual flea abundance of the most abundant species, *Ctenocephalides* spp., *Dinopsyllus* *lypusus* and *Pulex irritans*, was used during analysis. A GLMM model with negative binomial distribution with log link, for each model, weights of small mammals were treated as random effect. The model with lowest AIC was selected. All statistical analyses were performed in IBM Statistical Product and for Service Solutions (SPSS) Version 23. Statistical significance was set at *p* = 0.05.

## 3. Results

### 3.1. Small Mammals and Collected Fleas

A total of 302 small mammals including rodents and shrews were captured across the selected habitats (Table 1). *M*. *natalensis* was the most abundant species (54.4%, *n* = 163) followed by *Rattus rattus* (13.6%, *n* = 41) and *Crocidura* spp. (10.3%, *n* = 31) (Table 1). Females were captured in relatively high proportion (56.0%, *n* = 169) compared with males (44.0%, *n* = 133). A total of 271 fleas were collected throughout the study and the species were *Ctenopthalmus* spp., *Dinopsyllus* sp., *Xenopsylla cheopis*, *D*. *lypusus*, *Nosopsyllus incisus*, *Xenopsylla brasiliensis*, *P*. *irritans*, *Ctenocephalides felis* and *Echidnophaga gallicanea* (Table 2). Of these, the four most abundant species were *Ctenopthalmus* spp. (31.0%; *n* = 84), *P*. *irritans* (30.3%, *n* = 82), *D*. *lypusus* (28.8%, *n* = 78) and *N*. *incisus* (4.1%, *n* = 11) (Table 2).

**Table 1:** The number and relative abundance of small mammals captured across the study areas during the study.

Host species	Number	Relative abundance (%)
*Mastomys natalensis*	163	54.4
*Rattus rattus*	41	13.6
*Crocidura* spp.	31	10.3
*Lophuromys flavopunctatus*	13	4.3
*Praomys delectorum*	13	4.3
*Lophuromys kilonzoi*	10	3.3
*Grammomys* sp.	9	3.0
*Arvicanthis* *nairobae*	7	2.3
*Mus* spp.	7	2.3
*Acomys* sp.	4	1.3
*Lemniscomys striatus*	3	1.0
*Acomys wilsoni*	1	0.3
Total	302	100

**Table 2:** Flea abundance on rodent and shrew host species collected from different habitats across the selected areas.

		Total no. of fleas collected per host (no. of fleas/host examined)									
Host species	No. of hosts	Cts	Dnl	Dns	Xnb	Xnc	Ctf	Ecg	Nis	Pli	Total
*A*. *nairobae*	7	1	0	1	0	0	0	0	0	0	2
*A*. *wilsoni*	1	0	0	0	0	0	0	0	0	0	0
*Acomys* sp.	4	0	2	0	0	0	0	0	0	0	2
*Crocidura* spp.	31	0	0	0	0	0	0	0	0	0	0
*Grammomys* sp.	9	1	0	0	0	1	0	0	6	0	8
*L*. *flavopunctatus*	13	16	0	0	0	0	0	0	4	0	20
*L*. *kilonzoi*	10	17	1	1	0	0	0	0	0	0	19
*L*. *striatus*	3	5	0	0	0	0	0	0	0	0	5
*M*. *natalensis*	163	21	71	3	2	3	0	0	1	7	108
*Mus* spp.	7	0	1	0	0	0	0	0	0	6	7
*P*. *delectorum*	13	23	3	0	0	0	0	0	0	0	26
*R*. *rattus*	41	0	0	0	0	0	1	4	0	69	74
Total	302	84	78	5	2	4	1	4	11	82	271

*Note:* Fleas: Cts, *Ctenopthalmus* spp.; Dnl, *Dinopsyllus lypusus*; Dns, *Dinopsyllus* sp.; Xnc, *Xenopsylla cheopis*; Xnb, *Xenopsylla brasiliensis*; Nis, *Nosopsyllus incisus*; Pli, *Pulex irritans*; Ecg, *Echidnophaga gallicanea*; Ctf, *Ctenocephalides felis*.

### 3.2. Prevalence of Helminths

A total of 215 small mammals were infected with one or more than one genus of gastrointestinal helminth, hence amounting to the prevalence of 71.2% (95*%*CI = 65.7–76.2). Eight genera were found namely *Hymnelopis*, *Angiostrongylus*, *Trichuris*, *Capillaria*, *Physaloptera*, *Syphacia*, *Taenia* and *Nippostrongylu*s. The four most prevalent helminths were *Hymnelopis nana*, *Hymnelopis diminuta*, *Angiostrongylus* spp. and *Trichuris* spp. (Table 3). The majority of gastrointestinal helminths (more than 79% of the total prevalence) were found in *M*. *natalensis*, *R*. *rattus* and *Crocidura* spp. (Table 3). The most infested individuals were captured from crop fields, fallow lands, human dwellings and plantation forests, accounting for more than 92% of the overall prevalence (Table 4). Small mammals from plague endemic wards had a prevalence of more than 78% of the total prevalence compared with the plague nonendemic wards sampled (Table 4).

**Table 3:** Gastrointestinal helminths collected in small mammals from different habitats across the selected study areas in Mbulu, Lushoto and Morogoro Rural districts.

Total no. of infected species with gastrointestinal helminths (prevalence in %)												
Host species	No. of hosts	Hn	Hd	Strd	Sph	Ang	Tri	Pys	Sng	Cap	Ctf	Nip
*M*. *natalensis*	163	18 (6.0)	50 (16.6)	5 (1.7)	2 (0.7)	50 (16.6)	76 (25.2)	14 (4.6)	9 (3.0)	20 (6.6)	3 (1.0)	25 (8.3)
*R*. *rattus*	41	6 (2.0)	13 (4.3)	1 (0.3)	0 (0.0)	0 (0.0)	4 (1.3)	2 (0.7)	5 (1.7)	1 (0.3)	1 (0.3)	0 (0.0)
*Crocidura* spp.	31	13 (4.3)	6 (2.0)	0 (0.0)	0 (0.0)	0 (0.0)	8 (2.6)	0 (0.0)	1 (0.3)	2 (0.7)	0 (0.0)	0 (0.0)
*L*. *flavopunctatus*	13	5 (1.7)	6 (2.0)	1 (0.3)	0 (0.0)	2 (0.7)	2 (0.7)	0 (0.0)	2 (0.7)	0 (0.0)	0 (0.0)	1 (0.3)
*P*. *delectorum*	13	0 (0.0)	1 (0.3)	0 (0.0)	0 (0.0)	2 (0.7)	3 (1.0)	0 (0.0)	1 (0.3)	1 (0.3)	1 (0.3)	2 (0.7)
*L*. *kilonzoi*	10	3 (1.0)	6 (2.0)	0 (0.0)	0 (0.0)	0 (0.0)	0 (0.0)	0 (0.0)	0 (0.0)	0 (0.0)	0 (0.0)	0 (0.0)
*Grammomys* sp.	9	0 (0.0)	2 (0.7)	0 (0.0)	0 (0.0)	3 (1.0)	3 (1.0)	0 (0.0)	1 (0.3)	0 (0.0)	0 (0.0)	1 (0.3)
*A. nairobae*	7	0 (0.0)	0 (0.0)	0 (0.0)	0 (0.0)	1 (0.3)	2 (0.7)	0 (0.0)	0 (0.0)	0 (0.0)	0 (0.0)	2 (0.7)
*Mus* spp.	7	0 (0.0)	2 (0.7)	0 (0.0)	0 (0.0)	0 (0.0)	1 (0.3)	0 (0.0)	0 (0.0)	0 (0.0)	0 (0.0)	0 (0.0)
*Acomys* sp.	4	1 (0.3)	2 (0.7)	0 (0.0)	0 (0.0)	2 (0.7)	1 (0.3)	0 (0.0)	1 (0.3)	0 (0.0)	0 (0.0)	0 (0.0)
*L*. *striatus*	3	0 (0.0)	0 (0.0)	0 (0.0)	0 (0.0)	0 (0.0)	0 (0.0)	0 (0.0)	0 (0.0)	0 (0.0)	0 (0.0)	1 (0.3)
*A*. *wilsoni*	1	0 (0.0)	0 (0.0)	0 (0.0)	0 (0.0)	0 (0.0)	0 (0.0)	0 (0.0)	0 (0.0)	0 (0.0)	0 (0.0)	0 (0.0)
Total	302	46 (15.2)	88 (29.1)	7 (2.3)	2 (0.7)	60 (19.9)	100 (33.3)	16 (5.3)	20 (6.6)	24 (7.9)	5 (1.7)	32 (10.6)

*Note:* Helmiths: Hn, *Hymnelopis nana*; Hd, *Hymnelopis diminuta*; Strd, *Strongyyloides* spp.; Sph, *Syphacia* spp.; Ang, *Angiostrongylus* spp.; Tri, *Trichuris* spp.; Pys, *Physaloptera* spp.; Sng, *Strongyle*; Cap, *Capillaria* spp.; Ctf, *Cysticercus fasciolaris*; Nip, *Nippostrongylus* sp.

**Table 4:** Prevalence of gastrointestinal helminths infection in small mammals in selected plague‐endemic and nonendemic areas of Tanzania.

Variable	Categories	Total number	Infected	Prevalence (%)	*p*
Sex	Female	169	97	32.1	0.554
	Male	133	118	39.1	
Habitat type	Crop field	94	78	25.8	0.010 ^∗^
	Fallow land	104	73	24.2	
	Human dwellings	47	25	8.3	
	Plantation forest	31	23	7.6	
	Near natural forest	10	6	2.0	
	Bush	16	19	3.3	
Wards	Bwakila chini	59	33	10.9	0.020 ^∗^
	Kisaki	22	13	4.3	
	Manolo	43	36	11.9	
	Nahasey	55	40	13.2	
	Shume	73	57	18.8	
	Yaeda Ampa	50	36	11.9	
Host species	*M*. *natalensis*	163	130	43.0	0.004 ^∗^
	*R*. *rattus*	41	20	6.6	
	*Crocidura* spp.	31	21	7.0	
	*L*. *flavopunctatus*	13	10	3.3	
	*P*. *delectorum*	13	5	1.7	
	*L*. *kilonzoi*	10	9	3.0	
	*Grammomys* sp.	9	5	1.7	
	*A*. *nairobae*	7	3	1.0	
	*Mus* spp.	7	4	1.3	
	*Acomys* sp.	4	3	1.0	
	*L*. *striatus*	3	1	0.3	
	*A*. *wilsoni*	1	0	0.0	

^∗^Statistically significance difference.

The prevalence of *H*. *nana* varied across small mammal species (*X* ^2^ = 33.952, df = 11, *p* < 0.001) and habitats (*X* ^2^ = 12.701, df = 5, *p* = 0.26), but did not vary between host sex (*X* ^2^ = 0.316, df = 1, *p* = 0.574) and wards (*X* ^2^ = 7.582, df = 5, *p* = 0.181). The prevalence of *H*. *diminuta* varied across wards (*X* ^2^ = 33.928, df = 5, *p* < 0.001), but did not vary between host sex (*X* ^2^ = 0.037, df = 1, *p* = 0.847), host species (*X* ^2^ = 16.662, df = 11, *p* = 0.118) and habitat type (*X* ^2^ = 4.23, df = 5, *p* = 0.532). The prevalence of *Trichuris* spp. varied between host species (*X* ^2^ = 34.956, df = 11, *p* < 0.001) and habitat type (*X* ^2^ = 16.193, df = 5, *p* = 0.006), but did not vary between host sex (*X* ^2^ = 0.233, df = 1, *p* = 0.629) and wards (*X* ^2^ = 7.562, df = 5, *p* = 0.182). The prevalence of *Angiostrongylus* spp. varied across host species (*X* ^2^ = 38.788, df = 11, *p* < 0.001), habitat type (*X* ^2^ = 27.656, df = 5, *p* < 0.001), host sex (*X* ^2^ = 4.844, df = 1, *p* = 0.028) and wards (*X* ^2^ = 44.368, df = 5, *p* < 0.001).

### 3.3. Association Between the Prevalence of Helminths and Flea Abundance

This study documented significant association between the overall prevalence of gastrointestinal helminths and the overall flea abundance (*p* = 0.019). However, the helminth species specific prevalence of *H*. *nana*, *H*. *diminuta*, *Angiostrongylus* spp. and *Trichuris* spp. was not associated with flea species total abundance (*p* > 0.05). In contrast, the mean abundance of *Ctenopthal*mus spp. was significantly associated with the total gastrointestinal helminth prevalence, whereas the individual flea species mean abundance of *D*. *lypusus* and *P*. *irritans* were not (*p* > 0.05). The mean abundance of *Ctenocephalides* spp. and *D*. *lypusus* had no association with the prevalence of *H*. *nana*, *H*. *diminuta*, *Angiostrongylus* spp. and *Trichuris* spp. (*p* > 0.05). *P*. *irritans* mean abundance was significantly associated with the prevalence of *H*. *diminuta* (*p* < 0.05) but not that of *H*. *nana*, *Angiostrongylus* spp. and *Trichuris* spp. (*p* > 0.05).

### 3.4. PCV and Hb

The mean of PCV and Hb concentration for different host species is shown in Figures 2 and 3, respectively. The mean PCV and Hb of different host species provided in Table 5. The PCV varied across host species (*H* = 22.273, df = 12, *p* = 0.035), and the pairwise comparison revealed significant variations between the pairs of *Grammomys* sp. and *Lophuromys kilonzoi*, *Praomys delectorum*, *Lophuromys flavopunctatus*, *M*. *natalensis*, *R*. *rattus*, *Mus* spp. and *Lemniscomys striatus*. Also, between the pairs of *Crocidura* spp. and *R*. *rattus*, and between *Acomys* sp. and *M*. *natalensis* and *R*. *rattus*, Kruskal–Wallis test revealed no significant difference between the Hb of different species (*H* = 14.089, df = 12, *p* = 0.295).

**Figure 2:**
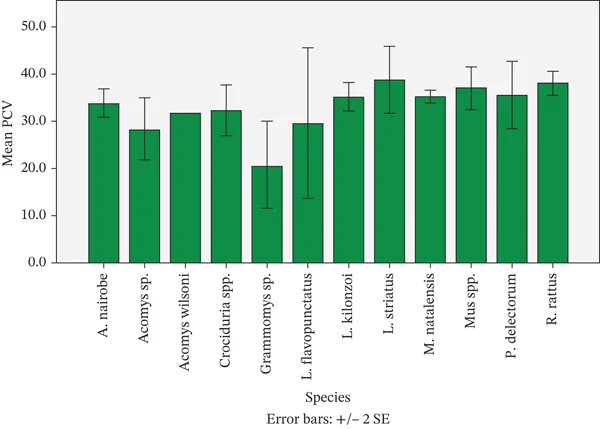
Mean PCV of small mammals collected from the study area.

**Figure 3:**
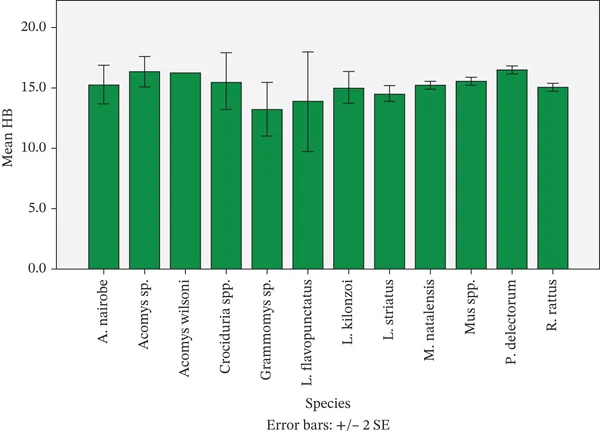
Mean Hb of small mammals collected from the study area.

**Table 5:** PCV (%) and Hb (g/dL) of different host species captured during study period.

Host species	PCV (*m* *e* *a* *n* ± *S* *E* *M*)	Hb (*m* *e* *a* *n* ± *S* *E* *M*)
*A*. *nairobae*	33.8 ± 1.6	15.3 ± 0.8
*Acomys* sp.	28.4 ± 3.3	16.3 ± 0.6
*Acomys wilsoni*	31.8 ± 0.0	16.3 ± 0.0
*Crocidura* spp.	32.4 ± 2.6	15.5 ± 1.2
*Grammomys* sp.	20.7 ± 4.6	13.3 ± 1.1
*L*. *flavopunctatus*	29.5 ± 8.0	13.9 ± 2.1
*L*. *kilonzoi*	35.1 ± 1.5	15 ± 0.7
*L*. *striatus*	38.7 ± 3.4	14.5 ± 0.4
*M*. *natalensis*	35.3 ± 0.7	15.2 ± 0.2
*Mus* spp.	37.1 ± 2.3	15.6 ± 0.7
*P*. *delectorum*	35.6 ± 3.6	16.5 ± 1.0
*R*. *rattus*	37.9 ± 1.2	14.9 ± 0.3

### 3.5. Association Between PCV and Hb Versus Flea Abundance

GLMM revealed that there was no significant association between PCV and Hb with flea species total abundance (*p* > 0.05). Furthermore, there was no significant association between PCV and Hb with individual flea abundance of *Ctenocephalides* spp., *D*. *lypusus* and *P*. *irritans* (*p* > 0.05).

## 4. Discussion

The present study investigated the effects of gastrointestinal helminths and haematological parameters (PCV and Hb) on flea abundance in rodents and shrews from selected areas of Tanzania. *M*. *natalensis*, *R*. *rattus* and *Crocidura* spp. were the most abundant small mammals captured across the different habitats. Similarly, *D*. *lypusus*, *Ctenophthalmus* spp. and *P*. *irritans* were the predominant flea species collected during the study period. These flea species have previously been reported as common in plague‐endemic foci in Tanzania and other regions [2, 32, 33]. The relatively high flea burden observed in *M*. *natalensis* and *R*. *rattus* may be attributed to their broad ecological tolerance and ability to adapt to diverse and adverse environmental conditions [34].

The overall prevalence of gastrointestinal helminths was 71.2%, with *H*. *nana*, *H*. *diminuta*, *Angiostrongylus* spp. and *Trichuris* spp. being the most prevalent taxa. Similar findings have been reported in other studies conducted in different geographical regions [6, 9, 12, 18]. Importantly, *H*. *nana*, *H*. *diminuta* and *Trichuris* spp. are zoonotic parasites that have frequently been reported in humans [35, 36]. Since *M*. *natalensis* and *R*. *rattus* commonly occur in and around human dwellings, the present findings highlight a potential public health concern in the study areas. The prevalence observed in this study was higher than that reported in central Iran (48.4%) [9] and slightly higher than that reported in Chile (68.5%) [37], but comparable to that reported in southwestern Iran (73.0%) [38]. Higher prevalences have also been reported in South Africa (86.7%) [39]. Within Tanzania, previous studies have similarly documented variation in gastrointestinal helminth prevalence across locations and seasons [6, 40]. The high prevalence recorded in the present study may be explained by the relatively large sample size and the timing of trapping, which coincided with the cropping season when food resources such as maize, beans and yams were abundant, thereby supporting high densities of small mammals [6, 40]. In addition, favourable environmental conditions, particularly temperature and humidity, may facilitate parasite development and persistence in the environment, and also the study was conducted during the wet season this might have some effect on the present findings as reported in Tanzania and elsewhere [6, 39].

Overall gastrointestinal helminth prevalence was significantly associated with total flea abundance. One possible explanation is that helminth‐infected hosts may experience reduced immune competence, making them more susceptible to flea infestation [16, 41]. Reduced immunity may weaken host defence mechanisms, including grooming behaviour, thereby increasing vulnerability to ectoparasite infestation. Furthermore, several flea genera, including *Xenopsylla*, *Pulex* and *Ctenophthalmus*, are recognised intermediate hosts for cestodes such as *H*. *nana* and *H*. *diminuta*, which were among the most prevalent helminths detected in this study. This may partly explain the observed association between *P*. *irritans* abundance and the prevalence of *H*. *diminuta*. In addition, many flea species are opportunistic and exploit a broad range of hosts, which may increase the likelihood of infestation in helminth‐infected small mammals [6, 13]. Coinfections are common in wildlife populations, and similar interactions between microparasites and macroparasites have previously been reported in *M*. *natalensis* populations in Tanzania [17]. In contrast, the prevalence of individual helminth taxa (*H*. *nana*, *H*. *diminuta*, *Angiostrongylus* spp. and *Trichuris* spp.) was not associated with overall flea abundance. This may be because the prevalence of individual helminth species was insufficient to explain variation in the combined abundance of all flea species.

The abundance of *Ctenophthalmus* spp. was significantly associated with the overall prevalence of gastrointestinal helminths. This may be explained by the broad host range of this flea genus, which has previously been reported to infest multiple small mammal species [5, 34, 42]. These findings further support the occurrence of polyparasitism in small mammals and suggest that interactions between endoparasites and ectoparasites may influence parasite community structure within host populations.

The high levels of parasitic infection and infestation observed in this study may also be influenced by the ecology and behaviour of small mammals. Many species frequently move between habitats in search of food and shelter, thereby increasing their exposure to infective stages of parasites [3]. In addition, the burrowing and predominantly nocturnal behaviour of many species increases contact with parasite‐contaminated nests and burrows [9, 18, 35]. Social behaviour may further facilitate parasite transmission because individuals often share shelters, promoting the circulation of infective stages within populations.

Significant variation in PCV was observed among small mammal species, whereas Hb concentration did not differ significantly. Similar variations in PCV have previously been reported in small mammals [43]. Several factors, including nutritional status, environmental conditions, habitat characteristics and behavioural adaptations such as fossorial or arboreal lifestyles, may influence haematological variation among species [43–45]. The lack of variation in Hb concentration may reflect similarities in environmental conditions experienced by these species, particularly among those sharing burrows and nests characterised by relatively low oxygen and elevated carbon dioxide concentrations. It is also important to recognise that parasitic and other infectious diseases may influence haematological parameters.

Neither PCV nor Hb was associated with overall flea abundance or the abundance of individual flea species. Although blood‐feeding ectoparasites are expected to benefit from hosts with higher nutritional blood quality, the present findings suggest that these haematological parameters alone are poor predictors of flea abundance. This contrasts with studies suggesting that host blood characteristics can influence vector fitness and reproductive performance [14, 15]. Several factors may explain this lack of association. First, there was considerable imbalance in sample sizes among host species. Second, many of the captured small mammals occupied similar habitats and were exposed to comparable environmental conditions, potentially reducing interspecific variation in blood characteristics. Third, most flea species are opportunistic rather than host‐specific and may infest a wide range of hosts regardless of blood nutritional quality [42, 46]. Finally, most of the captured rodents belonged to the family Muridae, which may have limited the range of physiological variation observed. Future studies should include a broader diversity of small mammal species to better understand the potential influence of haematological parameters on flea abundance.

## 5. Conclusion

This study demonstrates that coinfections involving gastrointestinal helminths and fleas are common among rodents and shrews in the selected areas of Tanzania. Overall gastrointestinal helminth prevalence was significantly associated with flea abundance, suggesting potential interactions between endoparasites and ectoparasites within small mammal hosts. In contrast, PCV and Hb were not associated with flea abundance, indicating that these haematological parameters alone may not adequately explain patterns of flea infestation. Given the important role of small mammals as reservoirs of zoonotic pathogens, these findings improve our understanding of host–parasite interactions and the ecological processes underlying polyparasitism. Future studies should incorporate larger sample sizes, broader taxonomic representation of small mammals and seasonal sampling to further elucidate the temporal dynamics and mechanisms linking gastrointestinal helminth infections, host physiology and flea infestation.

## Author Contributions

A.E.M. and L.L.M. conceptualised and designed the study; A.E.M. conducted field data collection, laboratory analyses, data analysis and drafted the initial manuscript; and L.L.M., J.S.N. and E.M.M. assisted with interpretations and conducted a series of reviews on the manuscript.

## Funding

This study was supported by the African Center of Excellence II through Innovative Rodent Pest Management and Biosensor Technology Development (IRPM‐BTD) (5799/TZ project).

## Disclosure

All authors have read and approved the submitted version of the manuscript.

## Ethics Statement

Ethical clearance was obtained from the Sokoine University of Agriculture (SUA) Ethics Committee (Ref. No. SUA/DPRTC/186/VOL IV). The permission to conduct this study across the selected areas of Mbulu, Lushoto and Morogoro Rural districts was obtained from the administrative authorities of respective districts.

## Consent

The authors have nothing to report.

## Conflicts of Interest

The authors declare no conflicts of interest.

## Data Availability

The datasets in this study can be provided upon reasonable request.
